# A feasibility study investigating the acceptability and design of a multicentre randomised controlled trial of needle fasciotomy versus limited fasciectomy for the treatment of Dupuytren’s contractures of the fingers (HAND-1): study protocol for a randomised controlled trial

**DOI:** 10.1186/s13063-017-2127-9

**Published:** 2017-08-25

**Authors:** Eleanor Harrison, Wei Tan, Nicola Mills, Alexia Karantana, Kirsty Sprange, Lelia Duley, Daisy Elliott, Jane Blazeby, William Hollingworth, Alan A. Montgomery, Tim Davis

**Affiliations:** 1 Nottingham Clinical Trials Unit, University of Nottingham, C Floor, South Block, Queen’s Medical Centre, Nottingham, NG7 2UH UK; 2 School of Social and Community Medicine, University of Bristol, Canynge Hall, 39 Whatley Road, Bristol, BS8 2PS UK; 3 Department of Academic Orthopaedics, Trauma and Sports Medicine, School of Medicine, University of Nottingham, Nottingham, NG7 2UH UK; 4 Queen’s Medical Centre, Nottingham University Hospitals NHS Trust, Derby Road, Nottingham, NG7 2UH UK

**Keywords:** Dupuytren’s contracture, Needle fasciotomy, Limited fasciectomy, Randomised controlled trial, Feasibility, Patient-reported outcome measure

## Abstract

**Background:**

Dupuytren’s contractures are fibrous cords under the skin of the palm of the hand. The contractures are painless but cause one or more fingers to curl into the palm, resulting in loss of function. Standard treatment within the NHS is surgery to remove (fasciectomy) or divide (fasciotomy) the contractures, and the treatment offered is frequently determined by surgeon preference. This study aims to determine the feasibility of conducting a large, multicentre randomised controlled trial to assess the clinical and cost-effectiveness of needle fasciotomy versus limited fasciectomy for the treatment of Dupuytren’s contracture.

**Methods/design:**

HAND-1 is a parallel, two-arm, multicentre, randomised feasibility trial. Eligible patients aged 18 years or over who have one or more fingers with a Dupuytren’s contracture of more than 30° in the metacarpophalangeal (MCP) and/or proximal interphalangeal (PIP) joints, well-defined cord(s) causing contracture, and have not undergone previous surgery for Dupuytren’s on the same hand will be randomised (1:1) to treatment with either needle fasciotomy or limited fasciectomy. Participants will be followed-up for up to 6 months post surgery. Feasibility outcomes include number of patients screened, consented and randomised, adherence with treatment, completion of follow-up and identification of an appropriate patient-reported outcome measure (PROM) to use as primary outcome for a main trial. Embedded qualitative research, incorporating a QuinteT Recruitment Intervention, will focus on understanding and optimising the recruitment process, and exploring patients’ experiences of trial participation and the interventions.

**Discussion:**

This study will assess whether a large multicentre trial comparing the clinical and cost-effectiveness of needle fasciotomy and limited fasciectomy for the treatment of Dupuytren’s contractures is feasible, and if so will provide data to inform its design and successful conduct.

**Trial registration:**

International Standard Registered Clinical/soCial sTudy Number: ISRCTN11164292. Registered on 28 August 2015.

**Electronic supplementary material:**

The online version of this article (doi:10.1186/s13063-017-2127-9) contains supplementary material, which is available to authorized users.

## Background

Dupuytren’s contractures are fibrous cords under the skin of the palm of the hand which typically occur in men and women over 50 years of age. They have a strong genetic tendency [1] and an increased incidence associated with diabetes and epilepsy [2]. The contractures are painless but cause one or more fingers to gradually curl into the palm, resulting in loss of hand function. This can lead to loss of dexterity for day-to-day tasks such as washing, grooming and shaking hands. It becomes increasingly difficult to put on a glove, hold large objects or put the hand in a pocket. Other difficulties experienced are diverse and patient dependent, and can include computer use, baking, piano playing, carpentry, gardening, and sports such as cycling, golf and tennis [3–5].

The standard treatment is surgery to remove (fasciectomy) or divide (fasciotomy) the Dupuytren’s contractures, allowing the finger to straighten better. However, this does not cure the condition, the finger may not come fully straight and recurrent contractures may require further surgery. In 2010–2011 it was estimated that the total cost of Dupuytren’s contractures procedures in England was more than £41 million with increasing rates of primary and revision procedures [6]. Increased longevity in an ageing population may cause a 77% increase in demand for treatment by 2030 [7].

There are no agreed criteria for choice of surgical treatment of Dupuytren’s contractures, but guidelines have been produced for one treatment, ‘needle fasciotomy’ (NF) [8]. The most common operation currently in the UK is ‘limited fasciectomy’ (LF), in which the fibrous cords preventing the finger from straightening are cut out of the hand through a long skin incision. This procedure is done under general or regional anaesthesia in an operating theatre, typically as a day-case admission and has a 4–6-week recovery period. Around 13,000 LFs were carried out in England in 2014–2015 [9]. A common alternative treatment is NF. In this procedure the fibrous cords preventing the finger(s) from straightening are divided with the sharp tip of a hypodermic needle which is passed through the skin without the need for a skin incision. It can be done in an outpatient clinic room and has a 1–2-week recovery period. Around 1300 NFs were recorded in UK Hospital Episode Statistics during 2014–2015, although this may be an underestimate if procedures performed in outpatient rooms are under-recorded [9].

In comparison to LF, NF is likely to be less expensive for health services, less disruptive for patients, and potentially carries a lower risk of complications that restrict hand function (temporarily or permanently) after the surgery [10]. Contractures can reform in the fingers that have been operated on after either treatment, causing the finger to bend up into the palm again, but recurrence occurs earlier and more frequently with NF, and may result in a need for further treatment [11]. There are three subtypes of Dupuytren’s contractures, those which affect the metacarpophalangeal (MCP) joint alone, the proximal interphalangeal (PIP) joint alone, or both the MCP and PIP joints. Both procedures can successfully straighten fingers with a Dupuytren’s contracture involving only the MCP joint. However, fingers with contractures involving the PIP joint cannot always be fully straightened with surgery, though LF is more successful and allows for the option of a PIP joint capsule release, if necessary.

There is no high-quality evidence comparing surgical treatments for Dupuytren’s contracture. One systematic review in 2010 found only five randomised or pseudo-randomised trials evaluating the surgical treatment of Dupuytren’s contracture [12], and a subsequent Cochrane systematic review found insufficient evidence to show the relative superiority of different surgical procedures (including NF versus fasciectomy) [13]. Although one small randomised controlled trial (RCT) comparing NF with LF showed a higher recurrence rate after NF at 5 years [11], it had methodological weaknesses, resulting in high risks of attrition, performance and detection bias [13], and did not rigorously compare hand function. Furthermore, recurrence should not be considered as indicating failure of the procedure, as it may not be sufficiently severe to warrant ‘revision surgery’ and may be managed by a further NF, with minimal inconvenience to the patient [14]. Also NF is cheaper and has a shorter recovery period than LF [10], and may produce as good hand function at 1- and 5-year follow-up [15].

Existing studies comparing NF with LF have a number of limitations; most importantly, high risk of performance and detection biases and the use of angular measurements of finger straightness and recurrence as primary outcomes rather than a patient-reported outcome measure (PROM) or an assessment of hand function. Also, none assessed the cost of treatments to either providers or patients, and none rigorously compared the outcomes of NF with LF in each of the three subtypes of Dupuytren’s contracture. The subtype, which only affects the MCP joint (40% of all contractures), is most likely to correct successfully with NF with the least risk of damage to the digital nerves and tendons [10, 11]. Surgeons are most comfortable treating this subtype by NF [16] and it may be a specific subgroup for which NF provides equivalent or better outcomes than LF.

Although function is the main problem for patients with Dupuytren’s contracture, there is no consensus about the most appropriate PROMs to assess the outcome of treatment. Most PROMs used in hand surgery are not specific to this condition. Thirteen small studies (four retrospective and six prospective cohort and three RCTs) have used PROMs as an outcome measure for Dupuytren’s contracture [17]. They used the DASH (11 studies), the QuickDASH, Part 2 of the Patient Evaluation Measure (PEM) and the Unité Rhumatologique des Affections de la Main (URAM) (one study each). Although improvements in hand function have been recorded with all after surgery, the minimal clinically important difference (MCID) for patients with Dupuytren’s contracture has only been calculated for the URAM, which may not adequately assess loss of hand function with Dupuytren’s contracture [5].

The lack of well-designed and well-conducted trials means that the choice of treatment for Dupuytren’s contractures of the fingers mainly depends on surgeon and patient preference. A survey of 116 hand surgeons showed marked variations in treatments advised for Dupuytren’s contractures [16]. The same surgeons reported that the most important research question about surgical treatment was a comparison of NF with LF. This is also an important question for patients. A recent survey [5] of 110 patients awaiting surgery found that the most important factors in deciding which treatment to undergo were recurrence (38%), surgeon guidance (37%) and speed of recovery following surgery (25%). There is an urgent need for robust evidence to guide decision-making.

In summary, a high-quality definitive trial comparing the outcomes and costs of NF with LF in patients with Dupuytren’s contracture of the MCP joint and/or PIP joint is needed. This study aims to assess the feasibility of a large multicentre trial, and to inform its design and conduct, including providing information about numbers of eligible patients, recruitment and randomisation, completion of follow-up, selection of appropriate outcome measures, and sample size.

### Objectives

The objectives of the study are to:

1. Define the eligibility criteria for a future, definitive randomised trial comparing NF with LF
2. Estimate the proportion of referred NHS patients with Dupuytren’s contractures who meet these eligibility criteria
3. Determine the willingness of surgeons to recruit patients with different patterns of Dupuytren’s contractures of the fingers
4. Estimate the proportion of eligible patients who consent to randomisation
5. Assess and optimise the recruitment process and patient pathway using a QuinteT Recruitment Intervention (QRI) [18]
6. Estimate follow-up and outcome completion rates
7. Evaluate outcomes for use as primary and secondary outcomes in the definitive study
8. Assess and compare validity and reproducibility of two methods of measurement of finger straightness which can be performed by a research assistant
9. Determine standard practice and equipment for clinic room provision of treatment
10. Assess the relationship between angular measurements of the finger deformity and patients’ reported outcomes
11. Evaluate the utility and acceptability of health resource use questionnaires to assess the impact of care on health service use and productivity
12. Assess patient and staff views on trial conduct, trial participation, and acceptability of interventions using qualitative research methods
13. Calculate the sample size required for a definitive study

## Methods/design

The HAND-1 study is a parallel, two-arm, randomised feasibility trial with participants individually allocated on a 1:1 ratio to treatment with either LF in an operating theatre, or NF in a clinic room. Participants will be followed up for up to 6 months post treatment. Embedded qualitative research incorporating a QRI [18] will focus on understanding and optimising the process of recruiting to the trial, and explore patients’ experiences of trial participation and the interventions. Audio-recordings of recruitment consultations will be a key method used to understand the recruitment process, along with regular monitoring of screening logs and interviews with trial staff and patients.

**Fig. 1:**
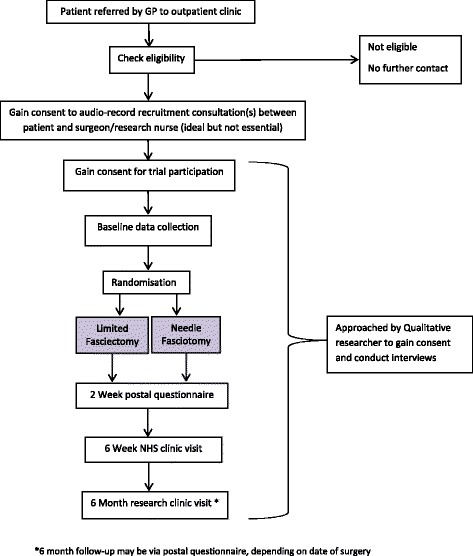
Trial flow diagram

Flow of participants through the study is summarised in Fig. 1.

The HAND-1 study will inform the design and conduct of a large multicentre trial to compare the clinical and cost-effectiveness of the two treatments.

### Participants

Recruitment will take place in three secondary care sites in England: Derby Teaching Hospitals NHS Foundation Trust, Wrightington, Wigan and Leigh NHS Foundation Trust and Nottingham University Hospitals NHS Trust/Nottingham Treatment Centre.

Patients are eligible for the study if they are aged over 18 years, have one or more fingers with a Dupuytren’s contracture of more than 30° in the MCP and/or PIP joints, have a well-defined cord causing contracture, have had no previous surgery for Dupuytren’s contracture on the same hand, are willing to undergo either study procedure, and are able to complete the follow-up assessments. Exclusion criteria are Dupuytren’s contracture of the distal interphalangeal joints (DIP) only, having a planned dermofasciectomy or very limited fasciectomy (excision of up to 1 cm of cord segment), previous recruitment into the study, or a life expectancy of less than 3 years.

### Interventions

The two interventions are NF and LF. Following randomisation, participants will be placed on the NHS waiting list for their allocated treatment, which will be carried out by a competent surgeon (consultant or experienced trainee, or an inexperienced trainee under direct supervision of their trainer). The level of experience of the surgeon and key elements of the procedure, including start and end times and equipment used, will be recorded.

#### Needle fasciotomy

The procedure will be performed in a clinic room (not an operating theatre) equipped with a good ‘clinic room’ spotlight, wound swabs, a couch and possibly an arm board. The hand will be rested appropriately to allow full extension of the affected finger (to put the cord under tension). The cord must only be cut and no segment must be excised. A small amount of local anaesthetic is injected at the site of each point of division of the cord. The number of points along the cord at which division is attempted and choice of needle size is at the discretion of the surgeon. The use of a knife is permitted, but no tourniquet or other surgical instruments will be allowed. Either a multiple stabbing technique or a side-to-side cutting action can be used to divide the cord(s) in as many places as indicated.

#### Limited fasciectomy

The planned operation must be a LF (excision of more than 1 cm of cord), and not a very limited fasciectomy or a dermofasciectomy. This will be performed under either general or regional anaesthetic with the use of a tourniquet. The contracture will be exposed through a standard surgical incision. For contractures involving the MCP joint the Dupuytren’s cord must be excised proximally to at least the proximal margin of the transverse fibres of the palmar aponeurosis. Digital cords should be excised completely from their origin. In all cases the distal margin of the cord excision should be the insertion of the cord onto the flexor sheath (or other structure). Deviations from ‘limited fasciectomy’ (for example, a decision made during surgery based on unexpected operative findings to use a skin graft) will be recorded, as will the use of additional procedures such as release of a joint contracture.

If a patient presents with two or more fingers on the same hand that require treatment, then both/all fingers will be treated in the same manner (i.e. both/all with LF or both/all with NF). For any study outcomes that require reference to a single finger, the finger which the patient reports pre-operatively as causing the most trouble will be used.

### Outcomes

#### Patient-reported outcome measures (PROMs)

A range of outcome measures were identified as being potentially useful for a large-scale trial, one of the main goals for this study being to determine their appropriateness and usability. These included validated questionnaires on physical function and symptoms, changes in wellbeing and health outcomes. Participants in both study arms will be asked to complete the same series of PROMs at baseline and at 2-week, 6-week and 6-month follow-up as follows:

1. Unité Rhumatologique des Affections de la Main (URAM) [19]
2. Disabilities of the Arm, Shoulder and Hand Questionnaire (DASH) [20, 21]
3. Part 2 of the Patient Evaluation Measure (PEM) [22]
4. Measure Yourself Medical Outcome Profile (MYMOP) [23]
5. EQ-5D-5 L descriptive system [24]

In addition, PEM will be completed on the day of surgery prior to treatment. This provides a check if symptoms have progressed differentially during this period, as waiting times from randomisation to surgery may differ for the two interventions.

#### Other clinical outcome measures

The following data will be collected at follow-up:

1. Global Improvement Item [23] – self-completed by participants (to act as the anchor for the assessment of the performance of the PROMs)
2. Complications following surgery
3. NHS resource use – patient-reported and obtained from medical records
4. Return to work/usual activities

The following objective outcomes will be measured at baseline and at 6-week and 6-month follow-up clinic visits:

1. Grip strength
2. Angular measurement of finger straightness, with photographs taken for blinded assessment

Standardised photographs will be taken using the Nottingham Dupuytren’s Assessor which has been developed for the purposes of the study.

### Sample size

As this is a feasibility study, a formal sample size calculation is not appropriate. It is anticipated that 50–85 participants will be recruited across the three sites. One of the primary aims of this feasibility study is to estimate response to invitation, eligibility, consent, randomisation and follow-up. Based on an expected total number to be invited of 400, estimated margins of error for these proportions will range between 5 and 13%.

### Recruitment and follow-up

Recruitment will take place from November 2015 to September 2016. Participants will be recruited from secondary care clinics at three sites in England. Patients who are referred by their general practitioner (GP) to the hand surgery outpatient clinic will be sent a short Patient Information Leaflet before their appointment which will explain Dupuytren’s contracture of the fingers and that they may be invited to participate in the study during their clinic visit. The leaflet will also explain that they may be asked for permission to audio-record consultations with the surgeon and research nurse/assistant during the clinic visit. These audio-recordings form part of the QRI and will help to understand and optimise the recruitment process. Trial posters will also be displayed in waiting areas in recruiting clinics to raise awareness of the study.

Following randomisation, participants are placed on the NHS waiting list for their allocated treatment, and each participant is followed up for 6 months following their surgery. At the end of the follow-up period information from the medical records will be extracted to record any outpatient appointments, outpatient procedures, emergency department visits or inpatient admissions related to the study hand in the 6 months after the initial procedure. Participants may be invited to take part in qualitative interviews up to 6 months after treatment.

**Fig. 2:**
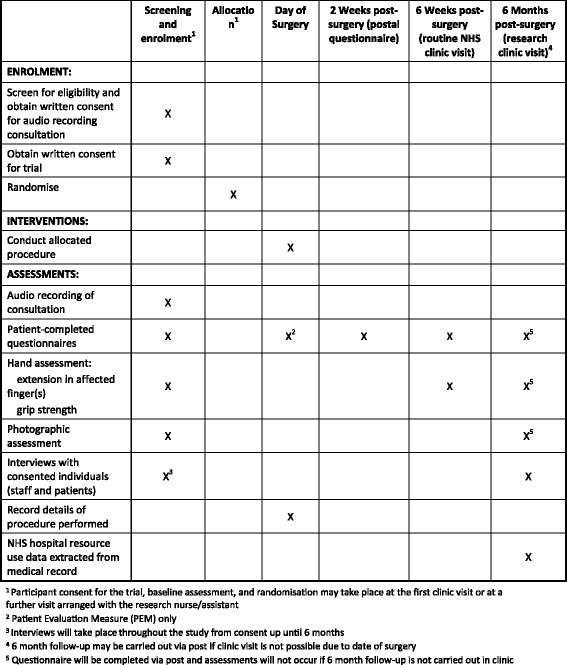
Schedule of data collection for the HAND-1 feasibility study (Standard Protocol Items: Recommendations for Interventional Trials (SPIRIT) figure)

Upon completion of all trial visits and questionnaires, participants will receive an end-of-study letter accompanied by an Information Sheet thanking them and informing them that their participation in the study is complete. Details of the data collection schedule are summarised (see Fig. 2).

### Consent

Participant Information Sheets (PIS) will be provided to patients and they will have the opportunity to discuss the study before agreeing to take part. Written informed consent will be obtained from all participants. Written consent for audio-recording consultations will be obtained separately from consent for the main study. The investigator, or their nominee, and the participant or other legally authorised representative will both sign and date the Informed Consent Form.

No trial-specific procedures will be conducted before informed consent has been obtained, and participants will be reminded that they may withdraw from the trial at any time without it affecting the quality of their care in the future.

### Randomisation

Participants will be randomly allocated to treatment, in a 1:1 ratio, to treatment with either LF in an operating theatre, or NF in a clinic room, via a secure web-based system which is maintained by the Nottingham Clinical Trials Unit (NCTU) in accordance with their standard operating procedures (SOPs). Randomisation will be stratified by recruiting site and the joints affected, and will use computer-generated, permuted, balanced blocks of randomly varying size. Investigators and research nurses will access the randomisation website by means of a remote, Internet-based randomisation system developed and maintained by the NCTU. Participants will be informed orally of their treatment allocation at the point of randomisation. Participants and their GPs will also be notified of the allocation by letter.

### Blinding

Blinding of the surgeon and/or participant is not possible as it will be clear which treatment the participant receives. Photographic evidence of contracture at the 6-month follow-up will be reviewed by an assessor blind to the treatment allocation, as the participant will be asked to wear a latex glove, with the tip for the study finger cut off, which will hide any surgical scars. Trial statisticians will be blinded to treatment allocation until database lock.

### Adverse events

Both interventions in the study are widely used within current NHS practice. As such, collection of adverse events (AEs), beyond those listed as trial outcomes, is limited to recording serious adverse events (SAEs). SAEs that will be reported are death, loss of a finger and any unexpected and serious event that is potentially related to the intervention. If site staff become aware of an SAE, they will complete an SAE Report Form which will be emailed or faxed to the NCTU within 24 h, and then forwarded to the medical monitor for assessment.

### Data collection, management and analysis

Data collection, clinical assessments and taking of digital images will be carried out by trained site staff. Participant questionnaires at baseline, day of surgery, 6 weeks and 6 months will be self-completed by participants during the clinic visit. Follow-up at 2 weeks will be via postal questionnaire.

If postal follow-up questionnaires are not returned, a telephone call will be made or a reminder letter sent from the NCTU to follow up with the participant. Participants who do not receive their allocated procedure will continue to be followed up unless they opt to withdraw from the trial completely. Participants who fail to attend the clinic visit at 6 weeks will still be invited to attend at 6 months unless they opt to withdraw from trial follow-up.

Data collection and retention rates will be monitored by the Trial Management Group (TMG) throughout the trial.

### Data management and monitoring

The NCTU will undertake data management and ensure that the trial is conducted according to Good Clinical Practice (GCP) guidelines and SOPs. Data will be collected and retained in accordance with the Data Protection Act 1998. Clinic data will be either entered directly into a web-based trial database at recruiting sites, or recorded on a paper worksheet and transcribed into the database, by site users with unique login details. Participant questionnaires completed at clinic visits will be transcribed by site staff into the trial database. Postal paper questionnaires will be returned in a reply paid envelope to the NCTU for data entry into the trial database.

Data quality will be ensured by database validation checks which include missing data, out-of-range values, illogical entries and invalid responses. Data entered by sites into the trial database will be subject to monitoring and review by NCTU staff, and data queries will be raised as necessary. Detailed data management processes and procedures are documented in the HAND-1 Data Management Plan.

Monitoring of study data will be by a combination of central and on-site monitoring, in accordance with the risk-based Monitoring Plan. The chief investigator (CI) has overall responsibility for the study and is custodian of the data.

### Statistical analyses

The analysis and reporting of the trial will be in accordance with extensions of the Consolidated Standards of Reporting Trials (CONSORT) Guidelines for Pilot and Feasibility Trials [25] and Non-Pharmacologic Treatment Interventions [26].

Continuous variables will be summarised in terms of the mean, standard deviation, median, lower and upper quartiles, minimum, maximum and number of observations. Categorical variables will be summarised in terms of frequency counts and percentages. A CONSORT flow diagram showing the numbers of people approached, eligible, recruited and randomised (with reasons for exclusions) will be produced. Recruitment rates at the start and end (after modification of the recruitment method based on the qualitative studies) of the recruitment phase of the study will be compared. Numbers and characteristics of participants recruited will be summarised using appropriate descriptive statistics and compared with patients who were eligible but not randomised. Completeness of data collection will be compared between trial arms. A Statistical Analysis Plan (SAP) will be agreed before data are unblinded.

We will ‘micro-cost’ [27] NF and LF by combining resource use with unit costs provided by the hospital finance departments. Standard unit costs will be used to estimate the NHS costs of care in the 6 months post procedure [28, 29]. Descriptive summaries of NHS cost data and return to work/usual activities at each follow-up time point will be presented.

Minimum clinically important effects for each PROM will be estimated using three anchor-based responsiveness statistics: (1) standardised response mean (SRM), (2) effect size (ES) and (3) Guyatt’s Responsiveness Index (GRI). This analysis will guide the choice of PROM for use in a definitive trial of treatment of Dupuytren’s disease, along with participant ranking of the different PROMs.

Improvements in the ability to extend the finger(s) after surgery will be compared with these responsiveness indices to investigate its use as a surrogate measure of hand function and calculate its minimum clinically important difference (MCID).

### Qualitative methods

Qualitative research will be integrated into the study to provide fundamental insights into the feasibility and design of a main trial. A QuinteT Recruitment Intervention (QRI) will be implemented to optimise trial recruitment [18], and further qualitative methods will be employed to explore patients’ experience of trial participation and the interventions.

#### QuinteT Recruitment Intervention

The QRI aims to understand the recruitment process and how it operates in each of the clinical centres. Sources of recruitment difficulties can then be identified and suggestions made to change aspects of the design, conduct, organisation or training that could then lead on to improvements in recruitment. The QRI will be flexible in its intensity and comprehensiveness to operate in the most effective way for the feasibility study. It will be undertaken in three distinct, but overlapping, phases.

Phase 1 seeks to understand the recruitment process as it occurs. A multifaceted, flexible approach will be adopted using one or more of the following methods until the point of data saturation – when new data does not materially add to the findings:

1. Monitoring the patient pathway through eligibility and recruitment
  All study centres will be asked to maintain detailed trial screening logs. This will record the details of patients who are, or are not, screened for trial entry, reasons for ineligibility and details of eligible patients who do not consent to trial participation and randomisation. These logs will be monitored regularly to identify patterns relating to recruitment rates, reasons for ineligibility, and points at which patients do not continue with trial recruitment.
2. Audio-recording of recruitment appointments
  All consultations in which the trial is discussed and the patient is offered participation in the trial will be audio-recorded following patient consent. The audio-recordings will be used to explore information provision, recruitment techniques, patient treatment preferences, and randomisation decisions to identify recruitment difficulties and improve information provision. The qualitative researcher will listen to appointments and document relevant details which will form the basis for individual confidential feedback and trial-specific training.
3. Semi-structured interviews
  Semi-structured interviews will be conducted with trial management and recruiting staff to assess their views on the trial and its conduct, including knowledge of the evidence and personal views about the interventions. Recruiters will also be asked how they explain the study to patients and perceived barriers to recruitment. Interviews may also be undertaken with a purposeful sample of up to 30 eligible patients soon after the offer of trial participation to explore their views on the trial and recruitment process, presentation of study information, study documentation and reasons for accepting or declining randomisation. A maximum variation sampling strategy [30] will be employed to ensure that a broad range of patients are captured. Interview topic guides will be used to ensure similar areas are covered in each interview within each group, based on those used in previous studies [31, 32], but will be sufficiently flexible to encourage the informants to express their own views about the study and any recruitment challenges expected or experienced.
4. Observations of investigator meetings
  Meetings between the CI, the Trial Management Group (TMG) and clinical investigators to discuss progress with the trial may be observed and audio-recorded to gather information about specific issues that may have a bearing on recruitment.
5. Study documentation
  Participant Information Sheets (PIS) and Consent Forms may be scrutinised to identify aspects that are unclear or potentially open to misinterpretation. They will be compared with the findings from the interviews and recorded appointments to identify any discrepancies or improvements that could be made.

In phase 2, the qualitative team will present summaries of anonymised findings from phase 1 to the CI and study management team, highlighting any factors that appear to be affecting recruitment with supporting evidence. It is likely that some aspects will be generic, such as how to explain randomisation and deal with patient preferences, as well as issues specific to the study. A plan of action will then be drawn up to optimise recruitment. This may include training sessions for recruiters, in which results are fed back and areas of difficulties addressed, recruitment tips documents, re-drafting study information to provide balanced information and changing aspects of organisation in clinical centres.

Numbers of eligible patients, and the percentages of these that are approached about the RCT, consent to be randomised and immediately accept or reject the allocation will be assessed in phase 3 before the plan of action is implemented, and regularly afterwards to check whether rates are improving. Follow-up interviews may also be conducted with the trial recruiters to ascertain their views on the acceptability of the QRI and any changes that may have occurred as a result.

#### Patients’ experience of trial participation and interventions

Semi-structured interviews will be undertaken with up to 30 trial participants within 6 months of receiving treatment to understand their experience with, and acceptability of, the treatment, outcome measures and wider trial processes. The final sample size will be driven by data saturation. Where possible, patients who were interviewed earlier on in the trial will be contacted again for this interview, or new patients will be purposefully sampled to ensure a broad range of participants. Topic guides will be used to ensure that similar topics are covered in each interview but applied in a flexible manner, enabling issues of importance to the patients to emerge. The guide will focus on their experiences of living with Dupuytren’s contracture pre and post intervention, previous experiences of treatment, recovery post intervention, views on the treatment received, the suitability and ease of understanding and completing the hand function outcome measures, and their reflections on participating in the trial.

#### Qualitative data analysis

Interviews and recruitment consultations will be audio-recorded, fully transcribed and, along with recruitment screening logs and observations, subject to simple counts, content, thematic and targeted conversation analyses. Preliminary analysis will be used to inform training and further data collection. Members of the qualitative team will independently analyse a proportion of transcripts to assess the dependability of coding, and will meet regularly to review coding and descriptive findings, agree further sampling and training strategies, and discuss theoretical development – all in close collaboration with the CI. Results from the qualitative research will help to inform the optimal design of a full-scale randomised trial.

#### Interim analyses

There are no planned interim between-group analyses. However, progress with recruitment and retention is monitored monthly by the TMG. If progress is below target, strategies will be implemented to improve progress in discussion with the TSC.

### Trial management and oversight

The trial co-ordinating centre will be the Nottingham Clinical Trials Unit (NCTU). Trial oversight will be by an independent Trial Steering Committee (TSC), which will meet at least twice a year and will provide an independent assessment of whether a full trial is feasible. As this is a feasibility study and both trial interventions are widespread clinical practice, there will be no independent Data Monitoring Committee.

The Trial Management Group (TMG) will meet monthly, and be responsible for the day-to-day management of the trial. Members of the TMG will report to the TSC at their meetings.

### Protocol amendments

The methods described in this protocol reflect the current study protocol (v 2.0 dated 21 June 2016). This protocol conforms to Standard Protocol Items: Recommendations for Interventional Trials (SPIRIT) recommendations (see Additional file 1 and Fig. 2). A summary of protocol amendments can be seen in Table 1. All amendments to the protocol and associated paperwork have been approved by the trial sponsor, Research Ethics Committee and local R&D departments prior to implementation.

**Table 1:** Summary of amendments to the HAND-1 feasibility study protocol

Protocol	Date	Summary of changes prior to start of recruitment
v 1.1	27 Aug 2015	• Minor administrative and typographical changes • Clarification that if a participant requires treatment on more than one finger, then both/all fingers will be treated in the same manner (i.e. both/all with limited fasciectomy or both/all with needle fasciotomy). For any study outcomes that require reference to a single finger, the one which the patient reports pre-operatively as causing the most trouble will be selected
Protocol	Date	Summary of changes after start of recruitment
v 2.0	21 Jun 2016	• Modification to 6-month follow-up so that participants who are unable to have a 6-month visit within the study follow-up period, because of the waiting time from randomisation to surgery, will be followed up via postal questionnaire only • Clarification that the qualitative research is carried out by researchers from the University of Bristol, and that participants may pause or stop audio-recordings or discussions with the qualitative researcher at any time • Clarification of serious adverse event (SAE) reporting timelines and process

### Confidentiality

Information about participants will be stored anonymously, confidentially and securely, and will be managed according to the requirements of the Data Protection Act, the NHS Caldicott Guardian and Research Governance Framework for Health and Social Care, conditions of REC approval and NHS information governance policy. Participant confidentiality will be ensured using unique identification numbers. Patient-identifiable information will be stored in locked filing cabinets in a secure room. Study data may be shared with the University of Bristol and other organisations as relevant where consent to do so has been obtained.

### Post-trial care

On completion of the study, participants will continue to receive routine NHS care as appropriate.

### Dissemination

The trial results will be reported in a peer-reviewed journal, and presented at scientific meetings. Reporting will be in compliance with CONSORT recommendations. Results will be made available to participants through a newsletter if they provide consent to receive this.

## Discussion

The current lack of robust evidence on treatment for Dupuytren’s contractures of the fingers means that the choice of treatment mainly depends on surgeon and patient preferences. A comparison of NF with LF has been identified as an important research question for both surgeons and patients [5]. However, it is uncertain whether surgeons will be willing to recruit, and whether patients will be willing to be randomised to such a study of two treatments which have very different patient pathways. Social circumstances, such as self-employment, duties as a carer for a relative and the financial burden of prolonged sick leave, may all influence each patient’s treatment preference, as may the desire for a straight, aesthetically satisfying finger, or to minimise the risk of needing further surgery in the future. Although some surgeons are confident with NF and willing to treat patients with well-defined cords causing either MCP or PIP joint contractures, others are reluctant to treat PIP contractures as they are concerned about damaging a digital nerve and also may not consider NF successful in treating these particular contractures. This is despite a large series reporting low incidences of digital nerve damage with NF [33] and a general acceptance that all procedures are less good at straightening PIP contractures than MCP ones. From a patient’s perspective, the options of NF and LF offer very different short- and long-term benefits, and thus many may find one treatment option suits their lifestyle much better than the other. The integrated qualitative and QRI component of the HAND-1 study will provide fundamental insights into the feasibility and design of a main trial.

The assessment of outcome of Dupuytren’s treatment with PROMs is in its infancy, and success or failure of the treatment has previously been determined by the amount of angular correction (straightening) of the flexed finger and the subsequent amount of recurrent angular deformity occurring over a pre-set time period, regardless of whether this results in the patient wishing to undergo further treatment to straighten the finger again. This is particularly unsatisfactory as the relationship between hand and finger function and joint-angle deformity is controversial.

The HAND-1 feasibility study will provide data essential to design and conduct a successful, future multicentre trial comparing the outcomes and costs of NF with LF for the treatment of Dupuytren’s contracture. This will provide robust evidence to guide clinical decision-making.

## Trial status

The HAND-1 study is ongoing. Recruitment commenced in November 2015 and is expected to continue until 30 September 2016.

## Additional file

Additional file 1: SPIRIT Checklist. (DOC 120 kb)

## Abbreviations

AE: Adverse event
DASH: Disabilities of the Arm, Shoulder and Hand Questionnaire
DIP: Distal interphalangeal joint
GCP: Good Clinical Practice
GP: General practitioner
GRI: Guyatt’s Responsiveness Index
LF: Limited fasciectomy
MCID: Minimal clinically important difference
MCP: Metacarpophalangeal joint
NF: Needle fasciotomy
NUH: Nottingham University Hospitals NHS Trust
PEM: Patient Evaluation Measure
PIP: Proximal interphalangeal joint
PIS: Participant Information Sheet
PROM: Patient-reported outcome measure
QRI: Qualitative Recruitment Intervention
R&D: Research and Development
RCT: Randomised controlled trial
REC: Research Ethics Committee
SAE: Serious adverse event
SOP: Standard operating procedure
SRM: Standardised response mean
TMG: Trial Management Group
TSC: Trial Steering Committee
URAM: Unité Rhumatologique des Affections de la Main Questionnaire
